# Silencing Smad7 potentiates BMP2-induced chondrogenic differentiation and inhibits endochondral ossification in human synovial-derived mesenchymal stromal cells

**DOI:** 10.1186/s13287-021-02202-2

**Published:** 2021-02-15

**Authors:** Pengcheng Xiao, Zhenglin Zhu, Chengcheng Du, Yongsheng Zeng, Junyi Liao, Qiang Cheng, Hong Chen, Chen Zhao, Wei Huang

**Affiliations:** Department of Orthopedics, The First Affiliated Hospital of Chongqing Medical University, Chongqing, 400016 China

**Keywords:** Human synovial-derived mesenchymal stromal cells, BMP2, Smad7, PPCNg, Chondrogenic differentiation, Endochondral ossification

## Abstract

**Background:**

Bone morphogenetic protein 2 (BMP2) is a promising chondrogenic growth factor for cartilage tissue-engineering, but it also induces robust endochondral ossification. Human synovial-derived mesenchymal stromal cells (hSMSCs) have attracted great interest due to their poor potential for differentiation into osteogenic lineages. Smad7 plays a significant in the endochondral ossification. In this study, we explored a new method to amplify the BMP2-induced chondrogenic differentiation of hSMSCs by downregulating Smad7 and applying a cellular scaffold.

**Methods:**

hSMSCs were isolated from human knee joint synovium from 3 donors through adhesion growth. In vitro and in vivo models of the chondrogenic differentiation of hSMSCs were established. Transgenic expression of BMP2 and silencing of Smad7 and Smad7 was achieved by adenoviral vectors. The osteogenic differentiation was detected by alkaline phosphatase staining, alizarin red staining, and RT-PCR analysis of the osteogenic genes RUNX2, Osterix, and Osteocalcin. The chondrogenic differentiation was detected by Alcian blue staining and RT-PCR analysis of the chondrogenic genes SOX9, COL2, and aggrecan. Hypertrophic differentiation was detected by the markers COL10 and MMP13. A subcutaneous stem cell implantation model was established with polyethylene glycol citrate-co-*N*-isopropylacrylamide (PPCN) scaffolds and athymic nude mice (3/group, 4–6 week-old female) and evaluated by micro-CT, H&E staining, and Alcian blue staining. An immunohistochemistry assay was used to detected COL1 and COL2, and an immunofluorescence assay was used to detect COL10 and MMP13.

**Results:**

These hSMSCs identified by flow cytometry. These hSMSCs exhibited lower osteo-differentiation potential than iMads and C3H10T1/2-cells. When Smad7 was silenced in BMP2-induced hSMSCs, the chondrogenic differentiation genes SOX9, COL2, and aggrecan were enhanced in vitro. Additionally, it silencing Smad7 led to a decrease in the hypertrophic differentiation genes COL10 and MMP13. In subcutaneous stem cell implantation assays, immunofluorescence and immunohistochemical staining demonstrated that silencing Smad7 increased the number of COL2-positive cells and decreased the expression of COL1, COL10, and MMP13.

**Conclusion:**

This study suggests that the application of hSMSCs, cell scaffolds, and silencing Smad7 can potentiate BMP2-induced chondrogenic differentiation and inhibit endochondral ossification. Thus, inhibiting the expression of Smad7 in BMP2-induced hSMSC differentiation may be a new strategy for cartilage tissue-engineering.

**Supplementary Information:**

The online version contains supplementary material available at 10.1186/s13287-021-02202-2.

## Introduction

The autologous repair capacity of the articular cartilage is very limited, because of the low mitotic activity of articular chondrocytes and the absence of the blood vessels to deliver putative precursor cells [1]. Tissue-engineering-based treatment strategies that include growth factors, seed cells, and implant scaffolds may open new avenues for the treatment of cartilage injuries [2, 3]. Many different growth factors, such as transforming growth factor beta (TGF-β), bone morphogenetic proteins (BMPs), and fibroblast growth factors (FGFs), have been shown to have the ability to promote stem cell chondrogenic differentiation [4, 5]. Among these factors, bone morphogenetic protein 2 (BMP2), a member of the TGF-β superfamily, has shown great potential for inducing the chondrogenic differentiation, osteogenic differentiation, and endochondral ossification of MSCs [6, 7]. As BMP2 has been approved by the United States Food and Drug Administration (FDA) and is widely used in spine surgery, its biosafety has been proven [8, 9]. Our previous study indicated that BMP2 is capable of promoting MSC chondrogenic differentiation, chondrocyte proliferation, and hypertrophic differentiation [10, 11]. A recent study by Chan et al. found that localized codelivery of BMP2 and soluble VEGFR1 (sVEGFR1) skewed the differentiation of resident skeletal stem cells towards the articular cartilage [12]. All of these studies suggested that BMP2-induced cartilage formation can be a promising solution for cartilage defect repair.

However, the use of BMP2 for bioengineered cartilage construction is still under debate, because BMP2 promotes not only chondrogenic differentiation but also endochondral ossification. Previous studies showed that hypertrophic markers, such as COL10 and MMP13, were expressed in the BMP2-induced ectopic cartilage-like mass at 21 days [13]. Vascular invasion, matrix calcification, and endochondral ossification were also observed in BMP2-induced MSCs, leading to the destruction of chondrogenic phenotypes [11, 14]. Therefore, endochondral ossification is the main obstacle to BMP2-induced cartilage formation [10, 11]. Thus, it is conceivable that BMP2 may be used for clinical cartilage repair if BMP2-induced chondrogenesis hypertrophy and endochondral ossification could be inhibited. To obtain a stable chondrogenic phenotype, many efforts have been made to inhibit the hypertrophic differentiation and endochondral ossification induced by BMP2.

Recently, human knee joint synovial-derived mesenchymal stromal cells (hSMSCs) have been reported to be one of the most ideal types of MSCs for cartilage regeneration research because they possess certain advantages, including the tissue specificity, abundant sources, powerful regenerative capabilities, and stable pluripotent differentiation potential [15, 16]. Mesenchymal stromal cells can easily be isolated from different tissues and stimulated by cytokines, leading to an upregulation of molecules typical of articular cartilage [17, 18]. In addition, it has been reported that synovium-derived stromal cells have relatively poor potential to differentiate into the osteogenic lineage in vitro [19, 20]. BMP2 has been demonstrated to govern the divergence of chondrogenesis in hSMSCs [17]. However, hSMSCs are also reported to be capable of endochondral ossification differentiation in vivo and thus can also impair chondrogenesis [18].

To further inhibit BMP2-induced endochondral ossification, we aimed to adjust the expression of Smad7 during cartilage formation. In our previous study, we found that overexpression of BMP2 leads to the upregulation of Smad7 expression [9]. Smad7, which belongs to the inhibitory Smad family, antagonizes the TGF-β/BMP signaling pathway through multiple mechanisms in both the cytoplasm and nucleus [21, 22]. Previous studies have shown that Smad7 is an inhibitor of BMP2-induced chondrogenic differentiation [23]. Moreover, Smad7 is essential for endochondral ossification [24]. In this study, we hypothesize that specific and stable BMP2-based tissue-engineered cartilage can be obtained by using hSMSCs with Smad7 expression knocked down. In addition, polyethylene glycol citrate-co-*N*-isopropylacrylamide (PPCNg), a biocompatible scaffold, was used to sustain cell attachment and proliferation [25]. We used hSMSCs infected with adenoviruses to investigate the effect of osteogenic and chondrogenic differentiation both in vitro and in vivo. We demonstrated that silencing Smad7 expression not only potentiated the chondrogenic differentiation induced by BMP2, but also inhibited the BMP2-induced hSMSC chondrogenesis hypertrophy. Therefore, our investigation provides another possible strategy for BMP2-based cartilage repair. These results will offer abundant practical evidence for BMP2-mediated bioengineered cartilage construction.

## Materials and methods

### Isolation and cell culture of hSMSCs

This research was approved by the Research Ethics Committee of the First Affiliated Hospital of Chongqing Medical University. All the participants were patients from the Orthopedic Department of the First Affiliated Hospital of Chongqing Medical University, aged from 25 to 40 years old who underwent lower limb amputations. We obtained informed consent from all three patients prior to their participation.

Primary hSMSCs were grown from human synovial membrane samples from the knee joint as previously reported [26]. Briefly, the human synovial tissue was washed with phosphate-buffered saline (PBS) containing 1% penicillin and streptomycin 3 times in a sterile petri dish and then minced with sterile scissors into 1–2 mm fragments. Two or three synovial fragments were then plated in 25 cm^2^ flasks in 5 mL of Dulbecco’s modified Eagle’s medium (DMEM, Gibco, China) containing 20% fetal bovine serum (FBS, Gibco, Australia) and 1% antibiotic-antimycotic solution (Gibco, China) and cultured for 14 days. When the cells grew to approximately 80% confluency, the synovial fragments were removed, and the cells were passaged into T25 flasks and considered as passage 0 (P0). In each experiment, the medium was refreshed every 3 days. When the cells reached 90% confluency, they were divided at a 1:2 ratio. In subsequent experiments, only cells between passages 1 and 5 were used.

### Cells culture

Immortalized mouse adipose-derived MSCs (iMADs) and C3H10T1/2 cells were obtained from ATCC (Manassas, VA). As previously noted [27], MSCs were cultured in DMEM with 10% fetal bovine serum (FBS), 1% penicillin, and streptomycin. The cells were grown in a monolayer under standard culture conditions (37 °C, 5% CO_2_).

### Crystal violet assay and CCK-8 assays

As reported [28], we seeded 10^4^ hSMSCs from passage 1 into each well of a 96-well culture plate and then cultured them for 11 days. At the indicated time-points, the cells were carefully washed with PBS and stained with 0.5% crystal violet/formalin solution at room temperature for 20–30 min. The stained cells were washed with tap water and air-dried before capturing macrographic images. Each time-point included six replicate wells. For quantitative measurement, the stained cells were dissolved in 10% acetic acid, and the optical density was measured at 590 nm as described [29].

To determine the change in cell proliferation ability with passaging, we used Cell Counting Kit-8 (CCK-8, MedChemExpress) to test hSMSCs at passages 1 and 5 [30, 31]. The results were recorded by a microplate reader (Thermo Scientific™, USA) at an absorbance of 450 nm. The growth curves were drawn, and the cell proliferation activity was analyzed.

### Phenotypic identification of hSMSCs

The identification of MSCs requires analyzing the expression of the relevant surface antigens [18]. hSMSCs were harvested and resuspended in PBS containing 1% bovine serum albumin at approximately 1 × 10^6^ cells/mL. Then, 0.1 mL of the cell suspensions was incubated with conjugated CD73 (BioLegend), CD105 (BioLegend), CD90 (BioLegend), CD45 (BD Biosciences), CD44 (BioLegend), HLA-DR (BD Biosciences), CD14 (BD Biosciences), or CD34 (BD Biosciences) antibodies for 30 min at 4 °C in the dark. After being washed three times with PBS, the labeled cells were resuspended in 0.2 mL of PBS and analyzed with the CytoFLEX system (Beckman Coulter). The acquired data were analyzed by using CytExpert software (Beckman Coulter) [26, 28].

Briefly, to identify the multiple differentiation potential [32, 33], cells were seeded in a 6-well plate, and the differentiation medium was replaced when the cell density reached 60%. Osteogenic medium was composed of DMEM with 10% FBS, 10 mM b-glycerophosphate (Sigma-Aldrich), 50 mg/mL ascorbate (Sigma-Aldrich), and 100 nM dexamethasone (Sigma-Aldrich). The osteogenic differentiation results were observed at 7 days with the BCIP/NBT Alkaline Phosphatase Color Development Kit (Beyotime) and at 21 days with Alizarin Red S staining (0.2%, pH = 8.3) (Solarbio). In terms of chondrogenic differentiation, we used Synovial Mesenchymal Stromal Cell Chondrogenic Differentiation Basal Medium (Cyagen) to induce hSMSCs for 14 days and assessed them by Alcian Blue staining (1%) (Solarbio) and immunohistochemical attaining of COL2 (Abcam, UK; 1:200). Adipogenic differentiation medium solution A consisted of DMEM complemented with 10% FBS, 10 mM dexamethasone, 50 g/mL indomethacin (Sigma), 45 mM 3-isobutyl-1-methylxanthine (Sigma), and 10 g/mL insulin (Sigma). Adipogenic differentiation medium solution B consisted of DMEM supplemented with 10% FBS and 10 g/mL insulin (Sigma). After incubation in solution A for 3 days, the medium was changed to solution B for another day. After the appearance of the morphologic features of differentiation, Oil Red O staining (0.5% in isopropanol) (Solarbio) was conducted to determine intracellular lipid droplet formation.

### Recombinant adenovirus construction

Recombinant adenoviruses were generated using AdEasy technology as described previously [10, 11]. The coding regions of BMP2, Smad7, red fluorescent protein (RFP), and green fluorescent protein (GFP) were amplified with PCR and cloned into adenovirus shuttle vectors. Then, the vectors were utilized to generate recombinant adenoviruses in HEK 293 cells [34]. The siRNA target sites against the human Smad7-coding region were cloned into the pSES adenovirus shuttle vector to create recombinant adenoviruses [35]. The resulting adenoviruses were designated Ad-GFP, Ad-BMP2, Ad-Smad7, and Ad-siSmad7. Ad-GFP and Ad-RFP were used as vector controls.

### RNA isolation and quantitative PCR (qPCR)

Total RNA was purified from cells in 60-mm dishes using TRIzol (Invitrogen) according to the manufacturer’s instructions [35]. Then, cDNA was obtained from total RNA extracted from cells using a reverse transcription (RT) reaction kit (TAKARA, Japan) [13, 36]. cDNA was completed with SYBR Premix Ex Taq™II (TaKaRa, Japan), and the program was carried out as follows: 95 °C for 30 s for one cycle; then, 95 °C for 5 s and 60 °C for 30 s, followed by plate reading for 40 cycles. The PCR primers (Table S1) were designed using Primer3 plus in Supplementary Table 1. The relative expression levels of the mRNAs in the groups were analyzed using the 2ΔΔCT method [32, 37].

### Western blot analysis

Cells were seeded in six-well plates and treated per the experimental design. Total protein was obtained after lysis, and the cleared lysates were denatured by boiling for 10 min with 10% sodium dodecyl sulfate-polyacrylamide gel electrophoresis buffer. The proteins were separated by electrophoresis with Tris-glycine gels and carefully transferred onto polyvinylidene difluoride (PVDF) membranes in the dark. Then, the PVDF membranes were blocked with 5% evaporated milk for 1 h and incubated overnight with primary antibodies against BMP2 (Abcam, USA) and Smad7 (Santa Cruz, USA). After washing, the membranes were probed with a fluorescently labeled secondary antibodiesy. The immune-reactive signals were detected using a Bio-Rad machine. In addition, the membranes were incubated with a monoclonal mouse anti-human β-actin (Abcam, USA) antibody as a loading control. The relative band intensity was measured using ImageJ analysis software [35, 36].

### Enzyme-linked immunosorbent assay

The concentration of BMP2 in the supernatants of infected hSMSCs was examined with enzyme-linked immunosorbent assay (ELISA) kits (Neobioscience, China). After 24 h of adenovirus infection, the supernatants of the two groups were collected and assayed according to the manufacturer’s instructions. The assays were performed in the same way after infection for 48 h and 72 h [32].

### ALP staining and activity

For ALP staining, cells were fixed with 4% paraformaldehyde for 30 min. The cells were then washed twice with PBS and stained using the BCIP/NBT Alkaline Phosphatase Color Development Kit (Beyotime). Staining was observed under a bright field microscope after 30 min [32, 38].

For the measurement of ALP activity, cells were washed twice with PBS and lysed with 150 μL of NP-40 lysis buffer (Beyotime). The cell lysates were quantified by an alkaline phosphatase assay kit (Beyotime) using p-nitrophenyl phosphate (pNPP) as the substrate. In the presence of magnesium ions, pNPP was hydrolyzed by phosphatases to phosphate and p-nitrophenol. The rate of p-nitrophenol liberation is proportional to ALP activity and can be measured photometrically. The ALP activity was measured by a microplate reader (Thermo Scientific™, USA) at an absorbance of 405 nm [39]. In addition, the protein concentration of the cell lysate was determined by a BCA Protein Assay Kit (Beyotime), and ALP activity was normalized to total protein per well.

### Matrix mineralization assay (Alizarin Red S staining)

Subconfluent hSMSCs, iMads, and C3H10T1/2 cells were infected with the indicated adenoviral vectors for 2 days. After adenovirus infection, the cells were cultured in the presence of ascorbic acid (50 mg/ml) and b-glycerophosphate (10 mM) for 14 days. The mineralization nodules were assessed by Alizarin Red S staining [28, 32]. Briefly, the cells were fixed with paraformaldehyde at room temperature for 10 min and washed with PBS (pH adjusted to 4.2). The fixed cells were incubated in a 37 °C incubator with 2% Alizarin Red S for 10 min, followed by careful washing with distilled water. The calcium deposits were observed under a microscope. For quantification, Alizarin Red S was dissolved in 10% acetic acid and the absorbance was detected at 405 nm with a microplate reader [32]. Total DNA was purified from cells per well using TRIzol (Invitrogen) and measured by spectrophotometer (Thermo, NanoDrop) [35]. The results were normalized to the total DNA per well and at least three independent experiments were performed.

### The chondrogenic and hypertrophic differentiation protocol

For the chondrogenic differentiation and hypertrophic differentiation of hSMSCs, the micromass culture method was used as previously noted [9, 10, 13]. Then, the cells were cultured in chondrogenic medium for 1 week, and this medium was composed of DMEM with ITS (Sigma-Aldrich), 50 μg/mL ascorbate (Sigma-Aldrich), 100 nM dexamethasone (Sigma-Aldrich), and 10 ng/mL transforming growth factor-β3 (Sigma-Aldrich). Then, the cells were cultured in hypertrophic medium for another week, and this medium contained ITS supplement, 50 μg/mL ascorbate, 1 nmol/L dexamethasone, and 100 ng/mL triiodothyronine (T3, Sigma-Aldrich) [39].

Briefly, hSMSCs were washed with PBS, treated with 4% paraformaldehyde for 30 min, and washed again with PBS, followed by staining with 0.5% Alcian blue in 0.1 M HCl (pH 1.0) for 12 h. Then, cells were photographed with a microscope [10, 11].

### Immunohistochemical analysis of cells

As previously reported [11], cell samples were fixed in 44% paraformaldehyde for 10 min and washed with distilled water. Next, the cells were treated with 3% H_2_O_2_ for 15 min at room temperature to eliminate the endogenous peroxidase activity and blocked with normal goat serum for 40 min at room temperature. Then, the cells were incubated with anti-Col2 (Abcam, UK; 1:200), anti-ColX (Santa Cruz Biotechnology; 1:200), and anti-MMP13 (Abcam, UK; 1:200) primary antibodies overnight at 4 °C. The sections were subsequently incubated with a secondary antibody, IgG-HRP (CST; 1:200). The resulting sections were photographed under a microscope.

### Stem cell implantation to ectopic cartilage formation and using of polyethylene glycol citrate-co-*N*-isopropylacrylamide (PPCN) scaffolds in vivo

Polyethylene glycol citrate-co-*N*-isopropylacrylamide (PPCN) was synthesized, as previously described [40]. PPCN powder was dissolved in PBS (at 100 mg/mL), sterilized by syringe filtration with 0.22 μm filters, and stored at 4 °C. Subcutaneous ectopic bone formation was carried out as previously reported [9–11].

Briefly, subconfluent hSMSCs were infected with specific adenoviruses until fluorescence could be seen and then harvested for subcutaneous injection. After washing with PBS, hSMSCs were resuspended in 5 × 10^6^ cells in 100 μL of PPCN scaffolds on ice. The cell/scaffold mixture was subcutaneously injected into the flanks of athymic nude mice (3 mice per group and 3 injections per mouse, 4–6 week-old female, the Experimental Animal Center, Chongqing Medical University, Chongqing, China). PPCNg alone and PPCNg with cells transduced with Ad-GFP, Ad-Smad7, or Ad-siSmad7 alone were used as controls. At 4 weeks, the animals were euthanized to harvest ectopic masses.

### Micro-computed tomography (μCT) analysis: hematoxylin & eosin (H&E) staining and Alcian blue staining

The retrieved masses were fixed with 10% PBS-buffered formalin and imaged using SkyScan1174 X-ray microtomograph (micro-CT) (Bruker Company, Belgian). NRecon software was used for 3D image reconstruction, and all the image data analyses were performed using CT-AN software. The total volume (TV) and bone volume/total volume (BV/TV) were measured as described [9, 32].

After μCT imaging, the retrieved masses were decalcified, paraffin-embedded, and sectioned. The sections were stained with H&E and Alcian Blue as previously described [9, 11].

### Immunohistochemical and immunofluorescence stain assays

In the immunohistochemical staining assay [41, 42], sections were deparaffinized with xylene, rehydrated using graded ethanol, treated with 3% H_2_O_2_ for 10 min to inhibit the endogenous peroxidase activity, boiled in citrate buffer (pH 6.0) for 20 min at 95–100 °C, and blocked with normal goat serum. Then, the sections were incubated with primary antibodies against COL2 (Abcam, 1:200) and COL1 (Collagen 1a1, Abcam, 1:200) at 4 °C overnight. After being washed, the sections were incubated with biotin-labeled secondary antibodies for 30 min, followed by incubation with streptavidin–HRP conjugate for 20 min at room temperature. Staining without primary antibodies was utilized as a negative control. All the images were obtained by using a microscope (Olympus).

In the immunofluorescence staining assay, the paraffin-embedded tissue sections were deparaffinized, rehydrated, and subjected to staining with an MMP13 antibody (Abcam, UK; 1:200) and Col10 antibody (Abcam, UK; 1:200) [32]. This was followed by incubation with corresponding fluorophore-conjugated antibodies. The immunohistochemical staining results were observed by inverted fluorescence microscopy (Olympus), and the images were analyzed using an Olympus auxiliary system.

### Statistical analysis

All the data are representative of at least three experiments performed in triplicate, which yielded similar results, unless otherwise indicated. All the quantitative experiments were performed in triplicate and/or repeated through three independent batches of experiments. The statistical analyses were performed using the software package SPSS 14.0 and by one-way analysis of variance and Student’s *t* test to determine the significance of differences between results, with *p < 0.05 and **p < 0.01 being regarded as significant [9, 10].

## Results

### Isolated hSMSCs that exhibit high proliferative activity

hSMSCs are considered to have the greatest potential for cartilage regeneration research due to their tissue-specific advantages. Although hSMSCs have been used rather extensively, their biological features are very different due to age, arthritis, or other donor joint conditions [18, 26]. Here, we isolated primary human synovial mesenchymal stromal cells from the knee joint synovial membrane of three donors.

**Fig. 1:**
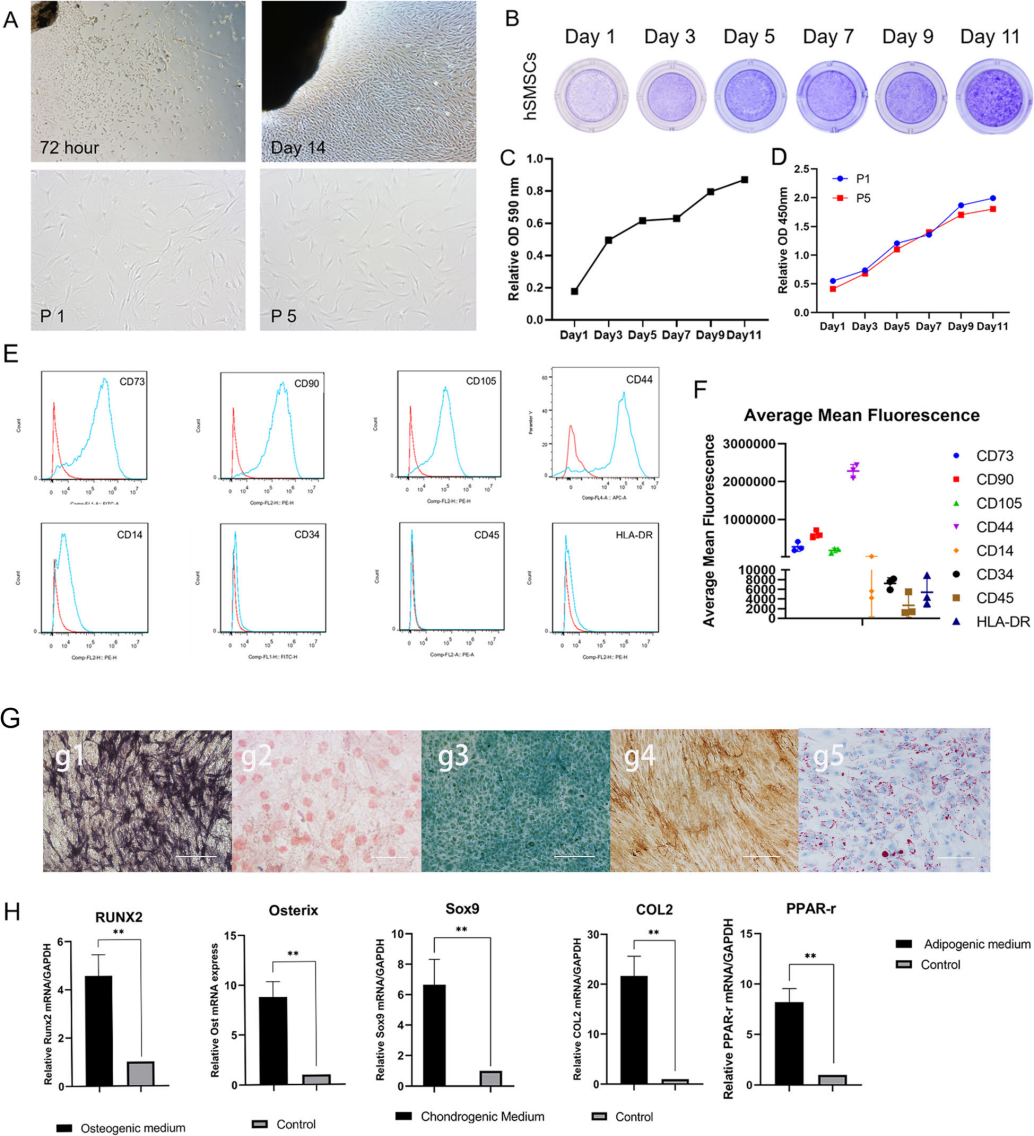
Isolation and morphology of cultured hSMSCs; the proliferation potential and the identification of hSMSCs. **a** hSMSCs were migrated outwards from the freshly harvested synovium at 72 h; Morphology of the confluence hSMSCs was recorded at day 14; primary cultures (P0) and the fifth passage (P5) with spindle shapes on cell culture dish (original magnification × 40, scale bar =200 μm) **b**. Cell proliferation assessed by crystal violet staining assay. General observation of the stained cells was recorded at the indicated time-points. **c** The stained cells were dissolved for quantitatively OD reading at A590 nm. **d** Proliferation of P1 and P5 as determined by the CCK-8 method showed that no significant difference. **e** The MSC markers of hSMSCs at P1 by flow cytometry and hSMSCs were positive for the MSC markers CD73, CD90, CD105, and CD44, while weakly expressed the markers CD34, CD14, CD45, and HLA-DR. **f** The average mean fluorescence for all markers between three donor populations. **g** The potential for osteogenic, chondrogenic, and adipogenic differentiation of hSMSCs in vitro: osteogenic (g1.alkaline phosphatase staining and g2.Alzarin Red S staining), chondrogenic (g3.Alcian Blue staining and g4.COL 2 immunohistochemical staining), and adipogenic (g5.Oil Red O staining) (original magnification × 200, scale bar = 50 μm). **h** RT-qPCR assay was performed to determine that the expression of osteogenic chondrogenic and adipogenic differentiation relative factors, including RUNX2, Osterix, Sox9, COL2, and PPAR-γ. The data are shown as mean ± SD for three separate experiments. *P < 0.05, **P < 0.01

After 72 h, fibroblast-like primary cells migrated outwards from the minced fragments of synovial tissues (Fig. 1a). Primary cells grew relatively slowly, and after 14 days of culture in dishes, the cells covered the field of vision (Fig. 1a). The morphology of cells cultured to passages 1 and 5 (P1 and P5, respectively) showed a spindle-shaped appearance and plastic-adherent properties, and there was no distinct change at passage 5 (Fig. 1a). Crystal violet staining assays indicated that hSMSCs reached 70% confluence at day 5, while at day 9, they covered the culture plate (Fig. 1b). Quantitative assessment of the stained cells showed that cell proliferation plateaued on day 5 and continued to increase after day 7 (Fig. 1c).

We used Cell Counting Kit (CCK)-8 to detect the effect of passage on hSMSC proliferative activity. The results showed similar growth capacities of P1 and P5 cells and confirmed the crystal violet staining results showing that cell proliferation had a short plateau period after day 5 (Fig. 1d). hSMSCs express most MSC markers and are capable of multidirectional differentiation potential.

According to the criteria for mesenchymal stromal cell identification, we identified the surface markers of hSMSCs using flow cytometry [29, 32, 33]. Flow cytometric results showed that P1 hSMSCs were positive for the MSC markers CD73, CD90, CD44, and CD105 and weakly expressed the hematopoietic markers CD34, CD14, CD45, and HLA-DR, which indicated that P1 hSMSCs express most of the consensus MSC markers and suggested that these cells may possess MSC-like characteristics (Fig. 1e). The average mean fluorescence for all markers between the three donor populations is shown (Fig. 1f).

In addition, when cultured in osteogenic, adipogenic, or chondrogenic medium, hSMSCs could readily be induced to differentiate into osteogenic, adipogenic, and chondrogenic lineages, respectively. Osteogenic potential was confirmed by staining with alkaline phosphatase (ALP) (Fig. 1g, g1) and Alizarin Red (Fig. 1g, g2). Chondrogenic potential was confirmed by staining with sulfated glycosaminoglycans using Alcian blue and COL2 protein using immunohistochemical staining (Fig. 1g, g3, g4), while adipogenic potential was evaluated by observation of small cytoplasmic lipid droplets stained using Oil Red O (Fig. 1g, g5). Osteogenic, chondrogenic, and adipogenic differentiation potential were also confirmed by RT-qPCR analysis of genes included the osteogenic relative factors RUNX2 and Osterix, the chondrogenic relative factors Sox9 and COL2, and the adipogenic relative factor PPAR-γ (Fig. 1h).

### Recombinant adenovirus effectively overexpressed transgenes in hSMSCs for a relatively long-time

**Fig. 2:**
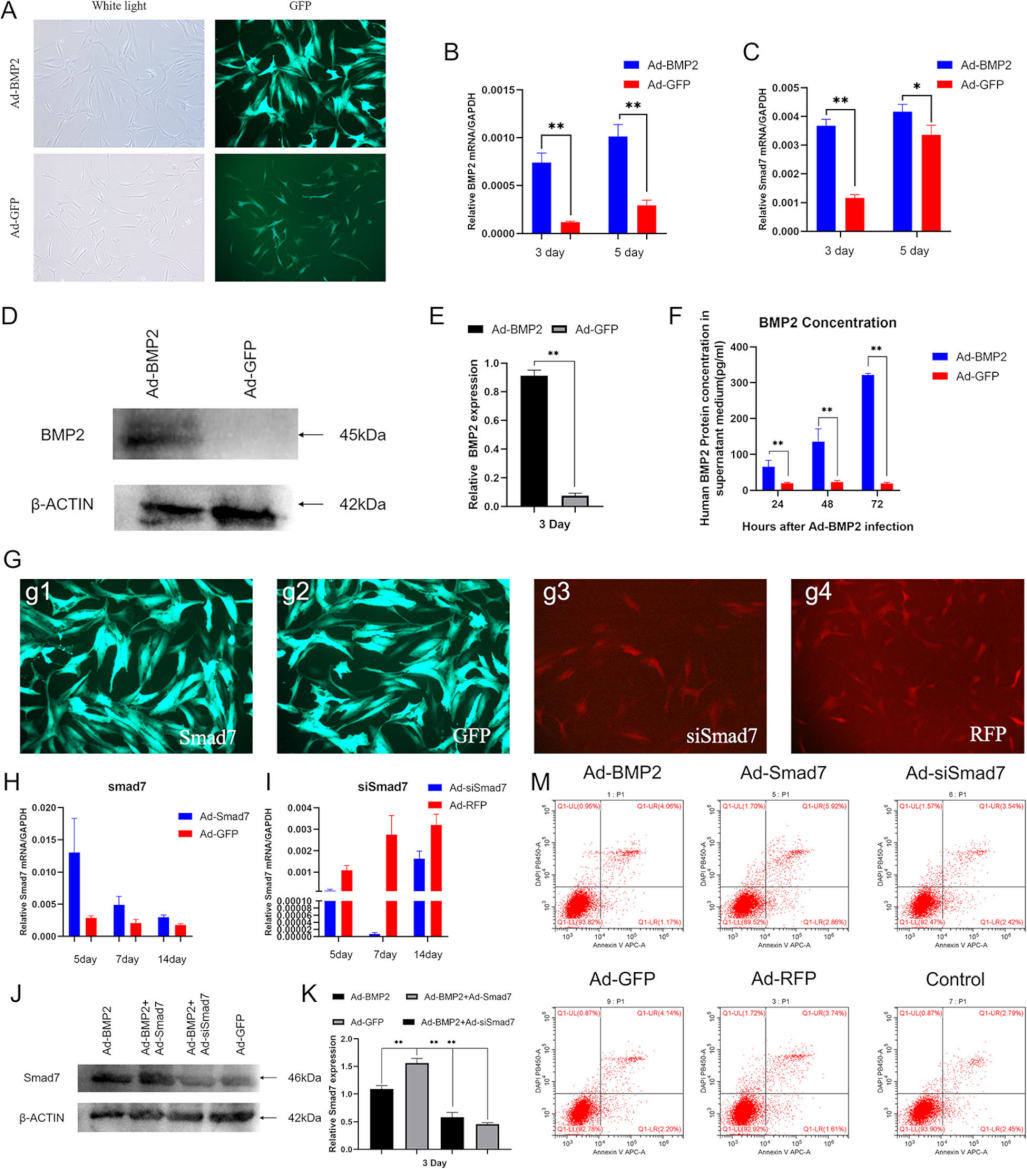
Adenovirus-mediated effective and safe transduction of BMP2, Smad7, siSmad7, RFP, and GFP into hSMSCs. **a** Representative bright field and GFP fluorescence fields were recorded at 24 h after infection (scale bar = 100 μm). **b** RT-PCR analysis of adenovirus-mediated transgene expression. Total RNA was isolated at 3 and 5 days after infection and subjected to RT-PCR using BMP2 primers. **c** RT-PCR analysis of Smad7 expression at mRNA level. BMP2 upregulated Smad7 mRNA levels at days 3 and 5 compared to the GFP group. **d** Western blot analysis of adenovirus-mediated transgene expression. Total cell lysate was collected from the hSMSCs at 72 h after infection and subjected to SDS-PAGE. The transgene expression of BMP2 was probed. Ad-GFP infected cells were used as negative controls. b-actin expression was used as loading controls. **e** The quantification results of the western blotting assay showed the increased expression level of BMP2. **f** Quantification of BMP2 protein levels on the medium supernatant by ELISA. Data were collected on 24 h, 48 h, and 72 h after adenovirus transduction. **g** The recombinant adenovirus Ad-Smad7 (green,g1), Ad-GFP (green,g2), Ad-siSmad7 (red,g3), and Ad-RFP (red,g4) were shown to effectively transfect hSMSCs for 48 h (scale bar = 100 μm). **h** Ad-Smad7 upregulated the expression of Smad7 at 5, 7, and 14 days. **i** Ad-siSmad7 silences the expression of Smad7 from 5 to 14 days. All samples were normalized with the house-keeping gene GAPDH. **j** Western blot analysis for the expression of Smad7 was conducted at day 3 after transduction of indicated recombinant adenoviruses (**k**), and quantitatively, relative Smad7 expression was analyzed and using b-actin as controls. **m** hSMSCs were infected with the indicated adenoviral vectors for 3 days. The infected cells were collected and subjected to apoptosis assay. Each experiment was done in triplicate. The data are shown as mean ± SD for triplicate. *P < 0.05, **P < 0.01

We constructed recombinant adenovirus to stabilize overexpression of BMP2 and Smad7 using AdEasy technology, and we constructed another recombinant adenovirus that expressed small interfering RNA (siRNA) targeting the coding region of human Smad7 using the recently described established pSOS system [9, 10, 13]. To determine whether recombinant adenovirus can be effectively transduced into hSMSCs, we observed the expression of GFP and RFP by fluorescence microscopy 24 h after infection (Fig. 2a). RT-PCR confirmed that the transgenes were highly expressed in the hSMSCs that were infected with the respective adenoviral vectors for 3 and 5 days (Fig. 2b). Moreover, we found that BMP2 effectively upregulated Smad7 expression at 3 and 5 days after infection (Fig. 2c). Western blot results showed that the Ad-BMP2 group effectively upregulated the BMP2 protein levels at 3 days (Fig. 2d,e). BMP2 is a secretory protein, and we performed ELISA to further detect the protein concentration in the cell supernatant [6]. The results showed that the BMP2 concentration in the supernatant increased gradually with increasing time (Fig. 2f).

To determine whether these recombinant adenoviruses could effectively regulate Smad7 expression levels in hSMSCs, fluorescence microscopy and RT-qPCR were used. The effectively transduction of adenoviral vectors was observed 48 h after infection (Fig. 2g). We found that Ad-Smad7 greatly upregulated the mRNA expression of Smad7 compared with the control (Fig. 2h). In addition, Ad-siSmad7 effectively knocked down Smad7 mRNA expression in hSMSCs from 5 to 14 days (Fig. 2i). At the protein level, western blot analysis revealed that the BMP2 and siSmad7 groups had lower expression levels of Smad7 than the BMP2 group and the BMP2 and Smad7 groups had higher expression levels than the BMP2 group (Fig. 2j, k).

MSC in vitro cultivation for clinical treatments may greatly affect MSC properties. A primary challenge is replicative senescence, which impairs MSC functions [43]. To protect the therapeutic potential of hSMSCs, we evaluated senescence after adenovirus transduction. After 72 h of adenovirus transfection, the cells were subjected to apoptosis assays (Fig. 2m) and beta galactosidase staining (Supplement, Figure 1), and no significant changes were observed compared with the control group.

### hSMSCs have less osteogenic differentiation potential in vitro

**Fig. 3:**
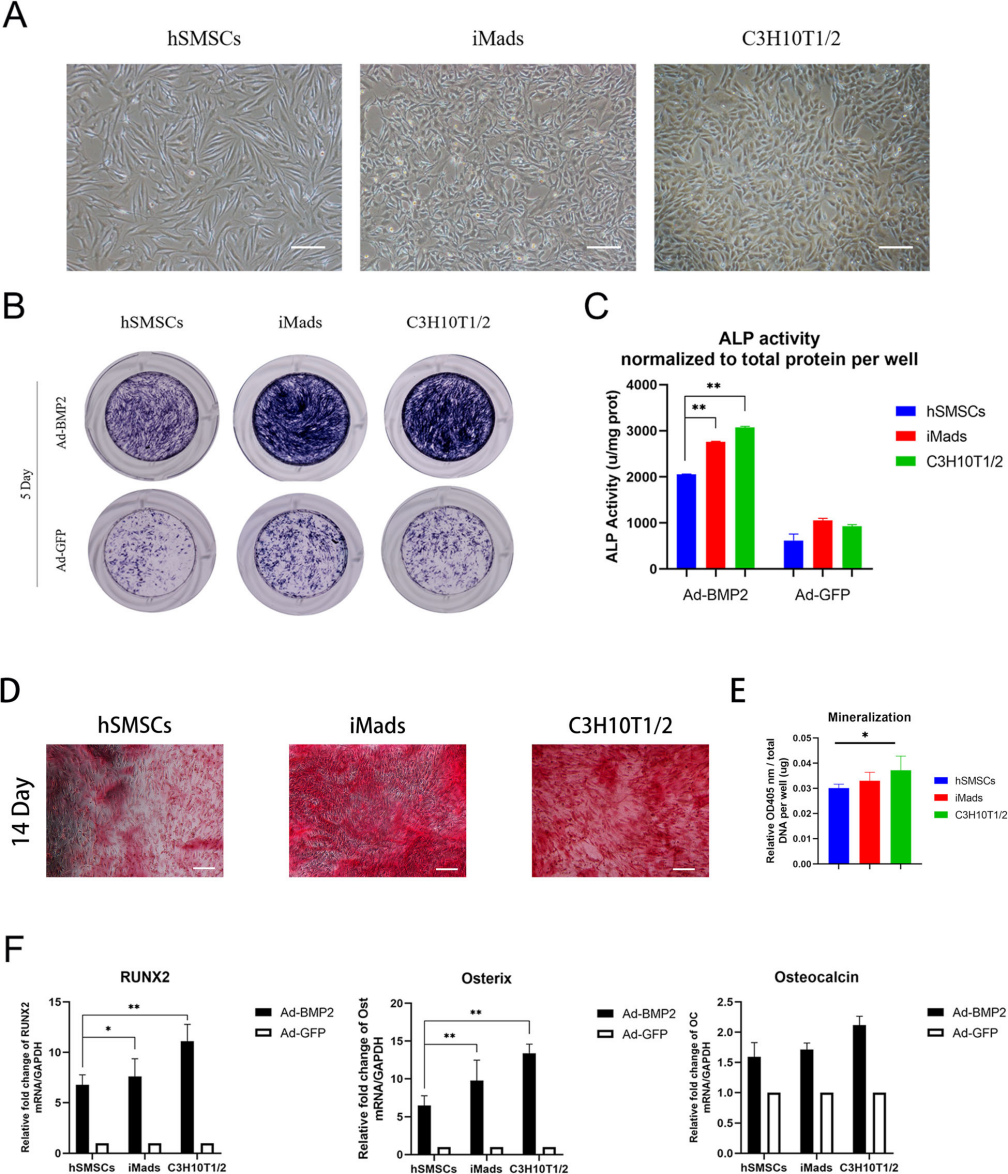
The hSMSCs has lower osteogenic differentiation potential in vitro. **a** Morphology of the confluence cells was shown (scale bar = 200 μm). **b** hSMSCs, iMads, and C3H10T1/2 cells were infected with AdGFP and AdBMP2. The ALP activity was measured at 5 days after transfection using ALP histochemical staining (Gross observation) (**c**). The ALP quantification activity was detected at OD 405 nm and normalized to total protein per well (unit/mg protein). **d** The mineralization and calcium deposition at the late osteogenic differentiation were observed by Alizarin Red S staining assay (scale bar = 100 μm) (**e**). Quantification results of Alizarin Red S staining showed that the mineralization effect in BMP2-induced hSMSCs was poor (day 14, normalized to total DNA per well, OD 405 nm/ug DNA). **f** RT-qPCR assay determined that hSMSCs have the lower expression level of osteogenic relative factors, including RUNX2, Osterix, and Osteocalcin at BMP2-induced osteogenic differentiation. Each experiment was done in triplicate. *P < 0.05, **P < 0.01

To assess the BMP2-induced osteogenic differentiation potential of hSMSCs compared with other MSCs, we used C3H10T1/2 cells and immortalized mouse adipose-derived MSCs (iMADs) (Fig. 3a). The cells were infected with the indicated adenovirus vectors. First, we examined the ALP activities by ALP staining at day 5, which indicated early osteogenic differentiation activity (Fig. 3b). Compared with that of hSMSCs, the ALP activity of C3H10T1/2 cells and iMads was considerably increased after BMP2 induction. Moreover, ALP quantitative analysis on day 5 showed that the ALP activity of the hSMSC group was dramatically lower than that of the other BMP2-treated groups (Fig. 3c). Second, Alizarin Red S staining was used to examine calcium deposition, which is one of the indicators of late osteogenic differentiation [6, 32]. The results showed that hSMSCs also had less calcium deposition than C3H10T1/2 cells and iMads at 14 days (Fig. 3d,e). Quantitative data of osteogenic-related gene expression detected by RT-PCR showed that RUNX2, Osterix, and Osteocalcin were significantly increased 7 days after BMP2 treatment (Fig. 3f). hSMSCs showed less osteogenic gene expression than other groups. Taken together, hSMSCs exhibit lower BMP2-induced osteogenic differentiation potential.

### Smad7 reduces BMP2-induced chondrogenic differentiation and enhances hypertrophic differentiation

**Fig. 4:**
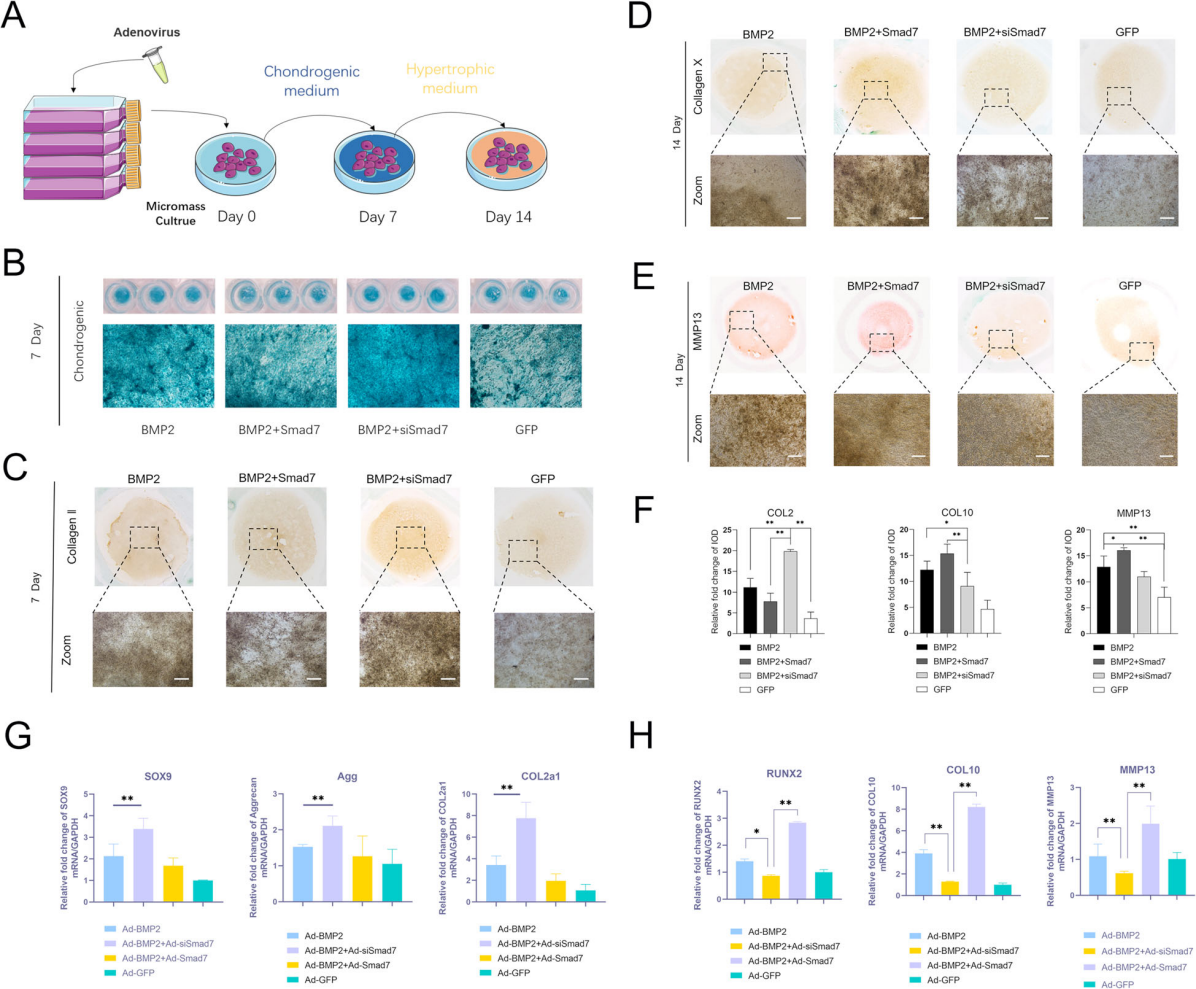
Smad7 reduce BMP2-induced chondrogenic differentiation and dilate hypertrophic differentiation. **a** The experimental design of Smad7 regulation during chondrogenic differentiation and hypertrophic differentiation. **b** Alcian blue staining for sulfated glycosaminoglycans in micromass cultures of hSMSCs on day 7 after transduction of indicated recombinant adenoviruses, gross observation, and microscope examination (40X) are shown. **c** Type II collagen (COL2) protein level was measured after 7 days by immunohistochemical analysis. Microscope bars was 100 μm. **d** COL10 protein level was measured after 7 days chondrogenic cultured and 7 days hypertrophic cultured by immunohistochemical analysis and both gross observation and microscope examination exhibits Smad7 promote its expression level. Bars 100 μm. **e** MMP13 protein level also measured by immunohistochemical staining. **f** Immunohistochemical staining quantification results were analyzed by ImageJ software. **g** RT-qPCR assay determined that Smad7 inhibits the expression of chondrogenic relative factors, including SOX9, Aggrecan, and COL2 at BMP2-induced chondrogenic differentiation. **h** Smad7 potentiates the expression of hypertrophic relative factors of hSMSCs. RT-qPCR assay was determined that hypertrophic differentiation relative factors, including RUNX2, COL10, and MMP13. All of the quantification data are done in triplicate and shown as mean ± SD. *P < 0.05, **P < 0.01

To confirm the function of Smad7 during BMP2-induced chondrogenic differentiation and hypertrophic differentiation during hSMSC chondrogenesis, we harvested adenovirus-infected hSMSCs and seeded them as micromasses; these micromasses were cultured under chondrogenic conditions for 7 days and then cultured under hypertrophic medium conditions for 7 days [10, 44, 45] (Fig. 4a). Alcian blue staining revealed that the level of sulfate glycosaminoglycans in the Smad7 silencing group was remarkably higher than that in the other groups and that in the Smad7 overexpression group was relatively lower than that in the BMP2 group on day 7 (Fig. 4b). Col2a1 is one of the most important molecular markers for chondrogenesis [2]. The immunohistochemical analysis results revealed that silencing Smad7 could significantly promote COL2 expression, while overexpressing Smad7 could inhibit BMP2-induced COL2 expression (Fig. 4c,f). The RT-PCR data revealed that the successfully transfected BMP2 groups had remarkably higher expression of chondrogenic marker genes, including Sox9, COL2, and Aggrecan, than the control group, while the chondrogenic marker gene expression in the silenced Smad7 group was most significantly increased during the chondrogenic differentiation process. Conversely, the chondrogenic marker gene expression in the Smad7 overexpression group was decreased compared with that in the BMP2 group (Fig. 4g).

To confirm the role of Smad7 in hypertrophic differentiation, Col X and MMP13 hypertrophic markers were detected by immunohistochemical staining [46]. The protein expression of Col X and MMP13 was clearly more upregulated in the BMP2 + Smad7 group at day 14 than in the other groups; the BMP2 + siSmad7 group showed lower protein expression levels than the BMP2 group and the BMP2 + Smad7 group but higher protein expression than the GFP group (Fig. 4d,e,f). Similarly, the RT-qPCR results revealed that the mRNA levels of RUNX2, Col X, and MMP13 in the BMP2 + siSmad7 group were significantly upregulated at day 14 compared to those in the BMP2 group (Fig. 4h).

In summary, these results indicate that Smad7 has a negative effect on chondrogenic differentiation-related factors and a positive effect on hypertrophic differentiation-related factors at the mRNA and protein expression levels.

### Silencing Smad7 expression promotes chondrogenesis of the ectopic PPCNg–hSMSC composite and inhibits endochondral ossification in vivo

**Fig. 5:**
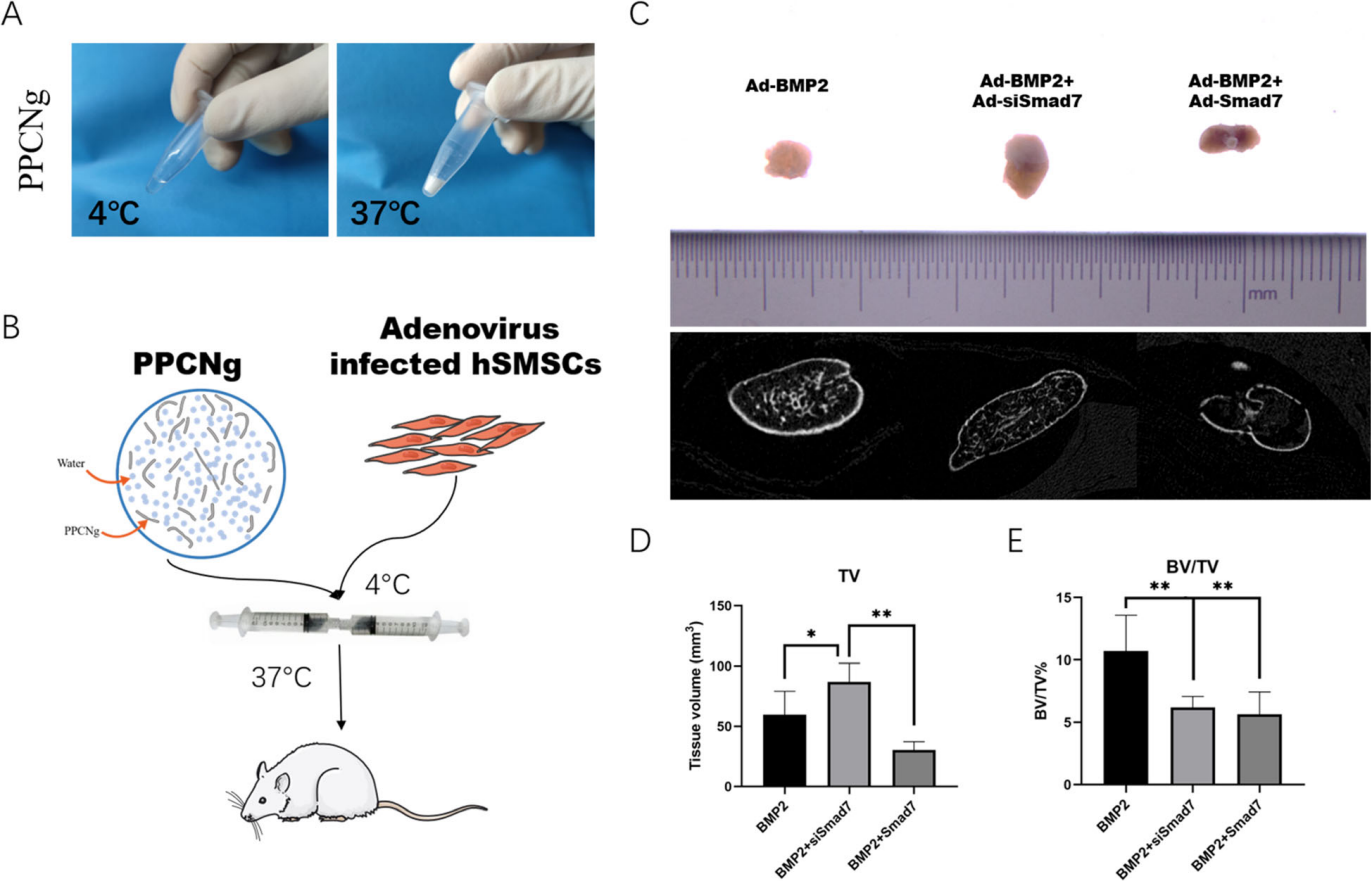
Silencing Smad7 enhances the BMP2 induced subcutaneous ectopic cartilage formation (**a**). Before and after thermal-induced gelation of PPCNg alone, respectively. **b** Illustrative diagram showing that hSMSCs treated as experimental design seeded on the scaffolds was implanted into the subcutaneous space of the nude mice and harvested for analysis after 4 weeks. **c** Macrographic images of ectopic masses. The micro-CT scanning to determine the surface and the volume of mass. **d** The retrieved masses were subjected to microCT analysis: Quantification results of the relative values of BV/TV were analyzed. These data are shown as mean ± SD repeat triplicate. *P < 0.05, **P < 0.01

We further examined the effect of combined PPCNg treatment on BMP2-induced chondrogenesis in vivo [9, 10, 13]. As a thermoresponsive macromolecule, PPCNg can provide a better in vivo microenvironment for hSMSCs [25]. The physical appearance of PPCNg remains liquid at 4 °C and quickly gels to form a solid scaffold at 37 °C [44] (Fig. 5a). We mixed adenovirus-infected hSMSCs and PPCNg at 4 °C and implanted them subcutaneously into nude mice (Fig. 5b). We found that PPCNg alone and PPCNg with cells transduced with Ad-GFP, Ad-Smad7, or Ad-siSmad7 alone failed to form any detectable masses (data not shown). The general observation and micro-computed tomography (micro-CT) results showed that compared with that in the BMP2 group, the volume of ectopic mass in the BMP2 + siSmad7 group was increased (Fig. 5c,d). Quantitative analysis of bone histomorphology showed that compared with the BMP2 + Smad7 group and BMP2 + siSmad7 group, the bone volume/total volume (BV/TV) in the BMP2 group was significantly increased (Fig. 5e).

**Fig. 6:**
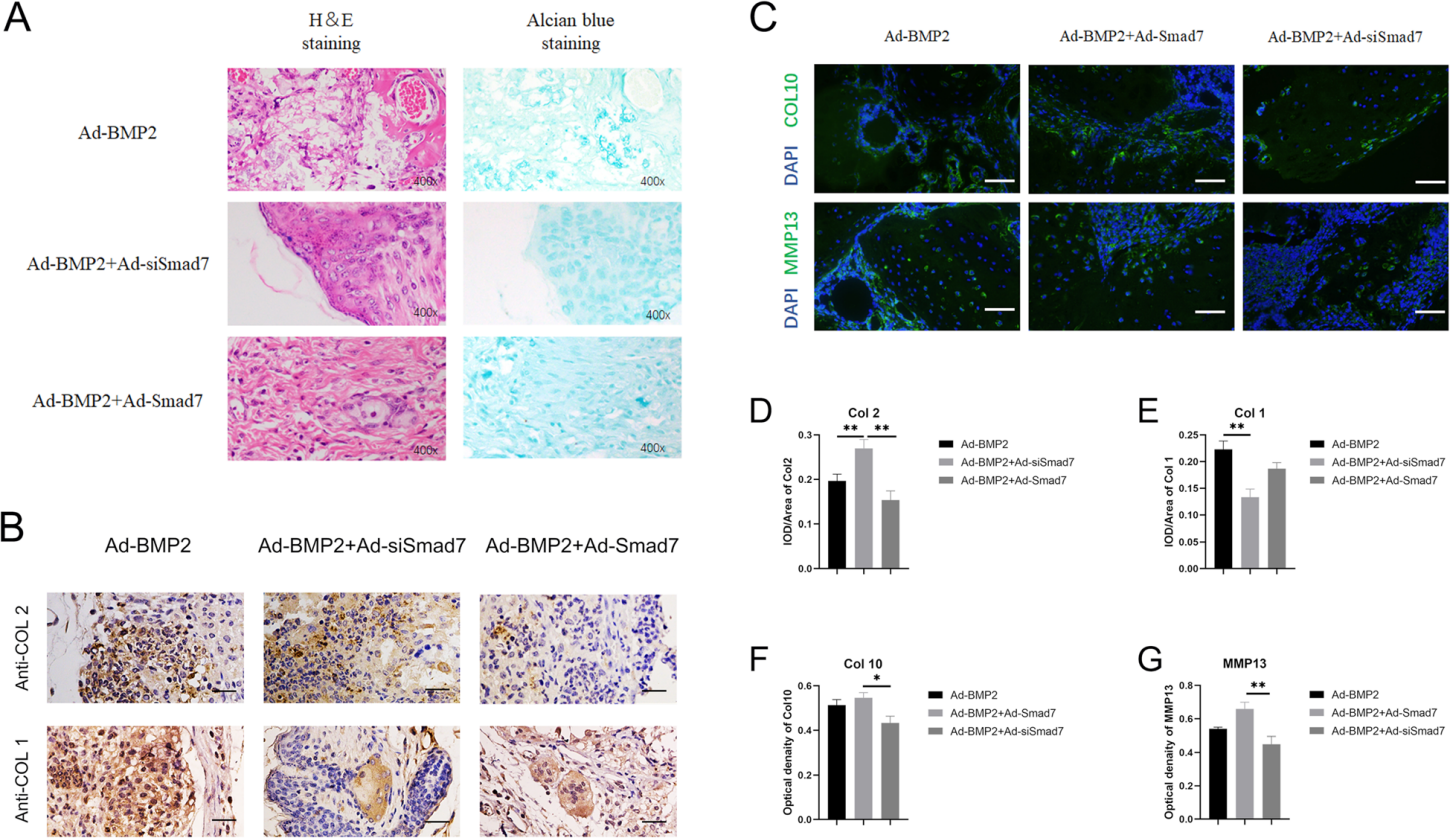
Histological analysis of the ectopic chondrogenesis. **a** The samples of ectopic masses were subjected to H&E staining and Alcian Blue staining to determine the formation of chondrocyte-like cells and hypertrophic chondrocyte-like cells (magnification × 400, scale bar =50 μm). Representative images are shown. **b** The immunohistochemical staining was utilized to confirm the influence of silencing of Smad7 in BMP2-induced chondrogenic differentiation in vivo. The expression of collagen type 1 (COL1) and Collagen type 2 (COL2) was detected (magnification of images × 400, scale bar = 50 μm) (**c**). Immunofluorescence staining was used to determine the effects of Smad7 on the expression of a hypertrophic marker, including COL10 (green) and MMP13 (green) and DAPI (blue) (magnification of up images = × 400, scale bar = 50 μm). **d**, **e** Quantitative analysis of positive immunohistochemical stained area. Integral optical density/area (IOD/Area) was calculated with ImageJ software. **f**, **g** Immunofluorescence staining quantified analysis were used optical density by ImageJ. *P < 0.05, **P < 0.01

Histological examination revealed that the BMP2 + siSmad7 group had more mature chondrocytes and a few hypertrophic chondrocytes (Fig. 6a). In the BMP2 group, trabecular bone and vessel invasion were detected, suggesting that endochondral ossification had already started. The Smad7 group showed a large amount of undifferentiated hSMSCs. In immunohistochemical staining, the BMP2 group stained positive for COL2 and the fibroblast marker COL1. The BMP2 + siSmad7 group also stained positive for COL2, and negative for COL1, suggesting that stable mature cartilage was formed (Fig. 6b, d, e).

Based on immunofluorescence staining, we found that MMP13 and COL10 were also decreased in the BMP2 + siSmad7 group (Fig. 6c,f,g). These in vivo findings further confirm the chondrogenesis-promoting effect of combined PPCNg and silenced Smad7 on BMP2-induced differentiation of hSMSCs.

## Discussion

BMP2 induces not only cartilage formation but also endochondral ossification, although the precise mechanisms remain to be fully understood [9, 10, 13]. Endochondral ossification is important for the development, growth, and repair of the long bones [2]. However, endochondral ossification is the main obstacle of cartilage tissue engineering because it damages the extracellular matrix of cartilage [11, 12]. BMP2-induced cartilage formation has been reported to be a possible strategy for cartilage repair. However, endochondral ossification during BMP2-induced differentiation still cannot be ignored and must be inhibited for further application of BMP2-mediated cartilage repair [9, 10, 13]. In this study, we aimed to achieve ideal chondrogenesis by improving three elements: the use of human synovial-derived MSCs, silencing Smad7 expression, and implanting injectable thermosensitive cell scaffolds.

Mesenchymal stromal cells can easily be isolated from different tissues, and in vitro stimulation by cytokines commonly leads to chondrogenic differentiation [18]. Compared with bone marrow-derived MSCs (BMSCs) and synovial-derived stromal cells exhibit relatively poor potential for osteogenic differentiation [19]. In recent years, De Bari et al. found that adult synovium-derived Gdf5-positive cells failed to mineralize in vitro but still had the potential for chondrogenic differentiation [47]. This result indicates that endochondral ossification is difficult in hSMSCs. In addition, hSMSCs possess certain advantages, including low immunogenicity in vivo and powerful regenerative capabilities in vitro [7, 20, 26, 27]. In the present study, primary human synovial mesenchymal stromal cells from the knee joint synovial membrane exhibited high proliferative activity and multidirectional differentiation potential. Moreover, these primary cells were identified by surface markers. However, we found that during BMP2-induced hSMSC differentiation in vitro, ALP activity was significantly increased. It also mineralized when cultured in osteogenic differentiation medium at day 14. The results suggest that BMP2-induced osteogenesis ability is still powerful in hSMSCs. In vivo, hSMSCs were also capable of endochondral ossification differentiation induced by BMP2. Even so, compared with adipose tissue-derived stem cells (iMads) and C3H10T1/2 mesenchymal stem cells of embryonic origin, hSMSCs have reduced osteogenic differentiation capacity. Our results indicate that among the most commonly studied MSCs, synovial stromal cells are the most specific for chondrogenic differentiation.

Smad7 may be the key to inhibiting endochondral ossification and affecting chondrogenesis [22, 23]. Our results revealed that Smad7 is upregulated in BMP2 overexpressing hSMSCs. As one of the two inhibitory Smad proteins, Smad7 is well known to inhibit TGF-β pathways in a variety of mechanisms [21, 48]. Previous studies showed that overexpressing Smand7 disturbed mesenchymal condensation decreased chondrocyte proliferation and inhibited chondrocyte maturation [22]. In the endochondral ossification process of embryonic chondrogenesis, Smad7 knockout mice showed both skeletal defects and shortened hypertrophic zones in growth plates [24]. On the other hand, the exogenous expression of BMP2 and Smad7 by adenovirus transfection decreased the length of the hypertrophic zone of the growth plate in fetal mouse tibia compared with transfected BMP2 only [9]. The effect of silencing Smad7 in BMP2-induced hSMSCs is still not reported. In this study, we used adenovirus transfection to significantly decrease Smad7 expression in hSMSCs to obtain a stable chondrocyte phenotype. In the chondrogenic differentiation experiment, silencing Smad7 expression significantly increased chondrogenic marker gene expression such as COL2a1 and SOX9, in hSMSCs. Furthermore, in the hypertrophic differentiation experiment, the Smad7-silenced group had lower expression of hypertrophic chondrocyte markers, such as Col X and MMP13, than the BMP2-induced only group. This result indicates that silencing Smad7 is a feasible strategy to increase chondrogenesis and matrix accumulation while inhibiting chondrocyte hypertrophic factor expression and endochondral ossification.

The cell attachment point and growth space are also indispensable for cartilage engineering [2, 3]. The subcutaneous transplantation of hSMSCs is limited by insufficient nutrient supply and irregular cell growth space, so we used PPCNg to improve this situation. This scaffold was mixed with a biodegradable citrate-based thermosensitive macromolecule, poly(polyethyleneglycol citrate-co-*N*-isopropylacrylamide) (PPCN), with gelatine (PPCN-g) [25]. The effectiveness of cell adhesion and the survival properties of MSCs have been proven by previous experiments in BMP9-induced osteogenic differentiation [40]. There are still no reports regarding the application of PPCN-g in MSC chondrogenic differentiation. We found that PPCN-g effectively promoted hSMSC chondrogenic differentiation in vivo. In ectopic hSMSC masses, the Smad7 silencing group had not only an increased number of COL2-positive cells but also a reduced number of hypertrophic chondrocytes and downregulated expression of the fibroblast marker COL1 and hypertrophic markers COL10 and MMP13. Compared with the BMP2 group, there was no obvious trabecular bone formation or new blood vessel invasion in the Smad7 silencing group. The standard endochondral ossification process is distinct from the in vitro experiment because of the lack of vascular invasion. Thus, the in vivo results have more practical value as a reference. The results strongly suggest that simultaneous application of hSMSCs, PPCN-g, and Smad7 knockdown can promote BMP2-induced chondrogenic differentiation and maintain the chondrocyte phenotype.

## Conclusion

In summary, our findings suggested that simultaneous application of hSMSCs, PPCNg, and Smad7 knockdown can promote BMP2-induced chondrogenic differentiation and maintain stable chondrocyte phenotype. Thus, it is conceivable that this method may be exploited as a novel strategy to treat cartilage injuries.

## Supplementary Information

**Additional file 1: Supplementary Table 1.** Primer sequence of the target genes. **Supplementary. Figure 1.** beta Galactosidase Staining of hSMSCs after adenovirus infection. **Supplementary Figure 3.** Angiogenesis of ectopic masses.

## Data Availability

The datasets used and/or analyzed during the current study are available from the corresponding author on reasonable request.

## Abbreviations

hSMSCs: Human synovial-derived mesenchymal stromal cells
BMP2: Bone morphogenetic protein 2
Smad7: SMAD Family Member 7
ITS: Insulin-transferrin-sodium
FBS: Fetal bovine serum
DMEM: Dulbecco’s modified Eagle’s medium
iMads: Immortalized mouse adipose-derived mesenchymal cells
PPCNg: Polyethyleneglycol citrate-co-*N*-isopropylacrylamide with gelatin
GFP: Green fluorescent protein
RFP: Red Fluorescent protein
CCK8: Cell Counting Kit-8
SOX9: SRY-Box Transcription Factor 9
COL1: Collagen type I
COL2: Collagen type II
COL2a1: Collagen type II alpha 1 chain
COL10: Collagen type X
MMP13: Matrix metallopeptidase 13
Agg: Aggrecan
ALP: Alkaline phosphatase
PBS: Phosphate-buffered saline
RUNX2: Runt-related transcription factor 2
