# Supplementary material for: Silencing Smad7 potentiates BMP2-induced chondrogenic differentiation and inhibits endochondral ossification in human synovial-derived mesenchymal stromal cells
Source: Stem Cell Res Ther. 2021 Feb 15;12:132. doi: 10.1186/s13287-021-02202-2 (PMC7885459; doi:10.1186/s13287-021-02202-2)

# Supplementary materials

**Silencing Smad7 improve BMP2-induced** **chondrogenic differentiation and inhibit endochondral ossification in human synovial derived mesenchymal stem cells**

Pengcheng Xiao^1^, Zhenglin Zhu^1^, Chengcheng Du^1^, Yongsheng Zeng^1^, Junyi Liao^1^, Qiang cheng^1^, Hong Chen^1^, Chen Zhao^1*^, Wei Huang^1*^

^1^ Department of Orthopedics, The First Affiliated Hospital of Chongqing Medical University, Chongqing 400016, China;

***Correspondence**

Wei Huang

Adress: Department of Orthopedics, the First Affiliated Hospital of Chongqing Medical University

Tel: +86-13883383330 Fax: +86 23 89011212

1. mail：huangwei68@263.net

Chen Zhao

Adress: Department of Orthopedics, the First Affiliated Hospital of Chongqing Medical University, Chongqing 400016, China

Tel: +86-13509428305 Fax: +86 23 89011212

E-mail：[Shawn95@yeah.net](mailto:Shawn95@yeah.net)

Supplementary. Table 1 Primer sequence of the target genes.

| SOX9-Forward | 5′‐AGCTCACCAGACCCTGAGAA‐3′ |
| --- | --- |
| SOX9-Reverse | 5′‐TCCCAGCAATCGTTACCTTC‐3′ |
| Smad7-Forward | 5′‐AAGATCGGCTGTGGCATC‐3′ |
| Smad7-Reverse | 5′‐CCAACAGCGTCCTGGAGT‐3′ |
| BMP2-Forward | 5′‐ACCAGACTATTGGACACCAG‐3′ |
| BMP2-Reverse | 5′‐AATCCTCACATGTCTCTTGG‐3′ |
| GAPDH-Forward | 5′‐CTATGAGGACCAGGTTGTCT‐3′ |
| GAPDH-Reverse | 5′‐TTGTCATACCAGGAAATGAGC‐3′ |
| RUNX2-Forward | 5′‐CCGGTCTCCTTCCAGGAT‐3′ |
| RUNX2-Reverse | 5′‐GGGAACTGCTGTGGCTTC‐3′ |
| COL2-Forward | 5′‐CAACACAATCCATTGCGAAC‐3′ |
| COL2-Reverse | 5′‐TCTGCCCAGTTCAGGTCTCT‐3′ |
| COLX-Forward | 5′‐GCATCTCCCAGCACCAGAAT‐3′ |
| COLX-Reverse | 5′‐GCTAGCAAGTGGGCCCTTTA‐3′ |
| MMP13-Forward | 5′‐TTGATGCCATTACCAGTCTCCG‐3′ |
| MMP13-Reverse | 5′‐CACGGGATGGATGTTCATATGC‐3′ |
| Agg-Forward | 5′‐TTGATGCCATTACCAGTCTCCG‐3′ |
| Agg-Reverse | 5′‐CACGGGATGGATGTTCATATGC‐3′ |
| Osterix-Forward | 5′‐TTCTGCGGCAAGAGGTTCACTC‐3′ |
| Osterix-Reverse | 5′‐GTGTTTGCTCAGGTGGTCGCTT‐3′ |
| Osteocalin-Forward | 5′‐CGCTACCTGTATCAATGGCTGG‐3′ |
| Osteocalin-Reverse | 5′‐CTCCTGAAAGCCGATGTGGTCA‐3′ |
| PPARγ-Forward | 5′‐AGCCTGCGAAAGCCTTTTGGTG‐3′ |
| PPARγ-Reverse | 5′‐GGCTTCACATTCAGCAAACCTGG‐3′ |

Supplementary. Figure 1 . beta Galactosidase Staining of hSMSCs after adenovirus infection.


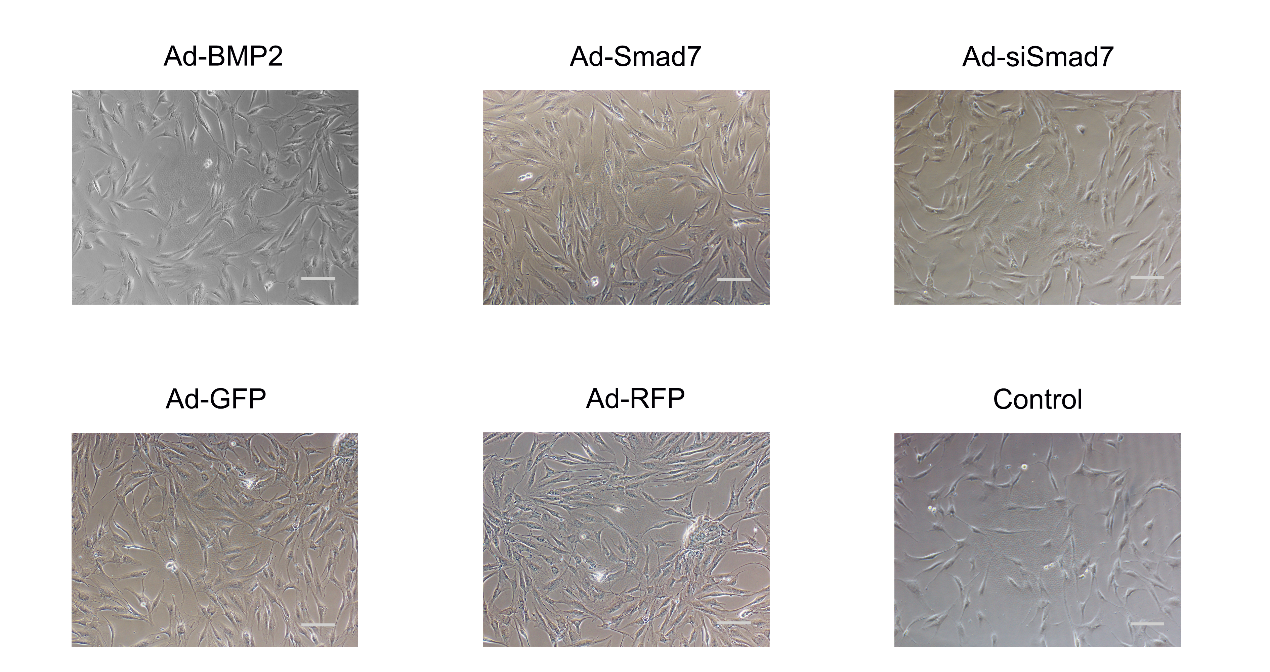


Supplementary. Figure 3 . Angiogenesis of ectopic masses.


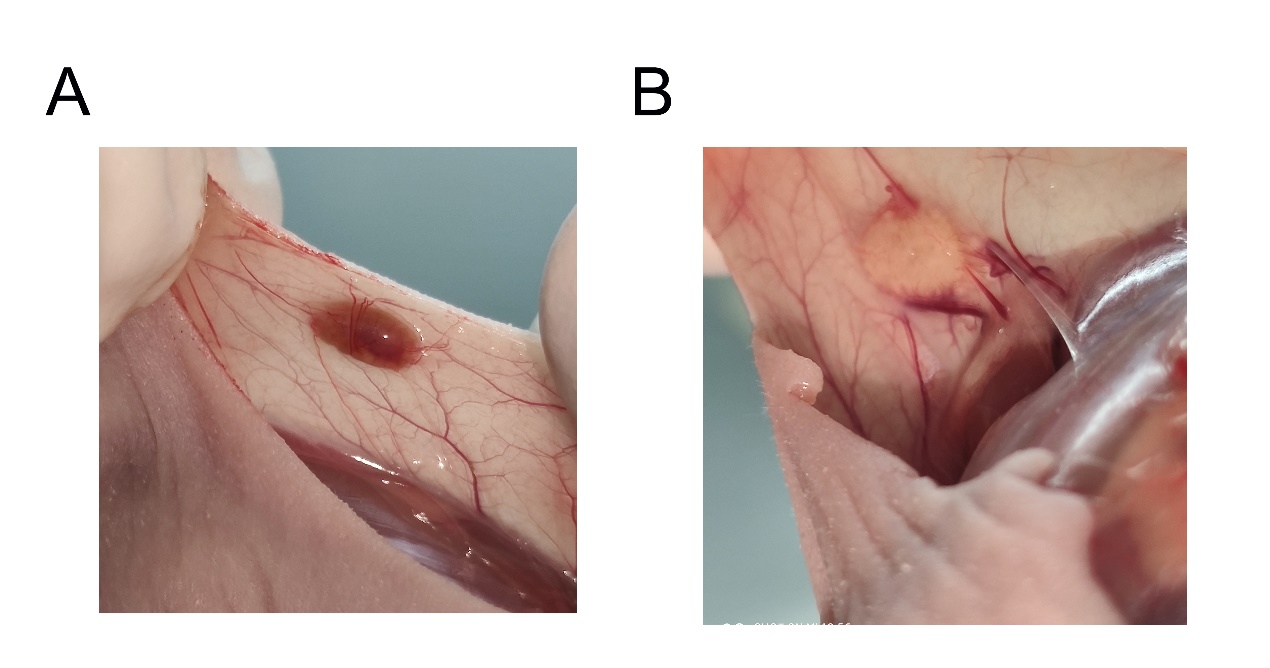

Supplement: Supplementary file 1 — Additional file 1: Supplementary Table 1. Primer sequence of the target genes. Supplementary. Figure 1. beta Galactosidase Staining of hSMSCs after adenovirus infection. Supplementary Figure 3. Angiogenesis of ectopic masses. [file 13287_2021_2202_MOESM1_ESM.docx]
